# Development and External Validation of a Novel Model for Predicting Postsurgical Recurrence and Overall Survival After Cytoreductive R0 Resection of Epithelial Ovarian Cancer

**DOI:** 10.3389/fonc.2022.859409

**Published:** 2022-03-23

**Authors:** Qiaqia Li, Yinghong Deng, Wei Wei, Fan Yang, An Lin, Desheng Yao, Xiaofeng Zhu, Jundong Li

**Affiliations:** ^1^ Department of Gynecologic Oncology, Sun Yat-sen University Cancer Center, Guangzhou, China; ^2^ State Key Laboratory of Oncology in South China, Collaborative Innovation Centre for Cancer Medicine, Guangzhou, China; ^3^ Department of General Surgery, Hunan Provincial People’s Hospital, The First Affiliated Hospital of Hunan Normal University, Changsha, China; ^4^ Department of Gynecology, Fujian Medical University Cancer Hospital, Fujian Cancer Hospital, Fuzhou, China; ^5^ Department of Gynecologic Oncology, The Affiliated Tumor Hospital of Guangxi Medical University, Nanning, China

**Keywords:** epithelial ovarian cancer, nomogram, predictive models, optimal resection, progression-free survival, overall survival

## Abstract

**Purpose:**

Treatment of epithelial ovarian cancer is evolving towards personalization and precision, which require patient-specific estimates of overall survival (OS) and progression-free survival (PFS).

**Patients and Methods:**

Medical records of 1173 patients who underwent debulking surgery in our center were comprehensively reviewed and randomly allocated into a derivation cohort of 879 patients and an internal validation cohort of 294 patients. Five hundred and seventy-seven patients from the other three cancer centers served as the external validation cohort. A novel nomogram model for PFS and OS was constructed based on independent predictors identified by multivariable Cox regression analysis. The predictive accuracy and discriminative ability of the model were measured using Harrell’s concordance index (C-index) and calibration curve.

**Results:**

The C-index values were 0.82 (95% CI: 0.76–0.88) and 0.84 (95% CI: 0.78–0.90) for the PFS and OS models, respectively, substantially higher than those obtained with the FIGO staging system and most nomograms reported for use in epithelial ovarian cancer. The nomogram score could clearly classify the patients into subgroups with different risks of recurrence or postoperative mortality. The online versions of our nomograms are available at https://eocnomogram.shinyapps.io/eocpfs/ and https://eocnomogram.shinyapps.io/eocos/.

**Conclusion:**

A externally validated nomogram predicting OS and PFS in patients after R0 reduction surgery was established using a propensity score matching model. This nomogram may be useful in estimating individual recurrence risk and guiding personalized surveillance programs for patients after surgery, and it could potentially aid clinical decision-making or stratification for clinical trials.

## Introduction

Ovarian cancer is the most lethal type of gynecologic malignancy, with an estimated 21410 new cases and 13770 deaths occurring in the United States in 2021 (1). The majority of these deaths are from epithelial ovarian cancer (EOC), which comprises 60% of ovarian cancers (2). Owing to the absence of specific symptoms and screening tools, EOC is usually diagnosed at an advanced stage, resulting in high rates of mortality and recurrence (3). Nowadays, the treatment of EOC is evolving towards personalization and precision, which require patient-specific estimates of overall survival (OS) and progression-free survival (PFS).

For first-diagnosed EOC patients, debulking surgery combined with platinum-based cytotoxic chemotherapy remains the cornerstone of standard management (4). Generally, an optimal debulking surgery is defined as ≤1 cm residual disease in the greatest dimension after resection (R0 resection), whereas >1 cm indicates a suboptimal surgery (R1 resection) (5, 6). Based on this, multiple predictive models have been established using R0/R1 as the main (sometimes only) prognostic variable describing surgeries (7, 8). However, several disconcerting facts have led to this practice being questioned. First, to maximize tumor removal, extensive resections including digestive tract resections or abdominal organ resections may be performed. Thus, “optimal resections” may comprise multiple surgeries that differ tremendously in the field of surgical complexity and long-term sequelae. A single index may be inadequate for describing such a set of surgeries (6, 9, 10). Second, numerous studies have proven that smaller amounts of residual disease are associated with longer survival, indicating that estimation of total tumor burden should be emphasized in predictive models (5). However, to the best of our knowledge, no predictive model has effectively quantified the total burden or refined the grey zone between R0 surgeries and complete resections. Therefore, it is natural to hypothesize that surgical complexity and total tumor burden are predictive factors of OS and PFS and should be taken into consideration in order to construct accurate predictive models.

Nomograms are statistical models that condense diverse prognostic variables into user-friendly pictorial representations to estimate the probability of a clinical event, e.g., recurrence or death, for an individual patient (11–13). As we aimed to devise an applicable and reliable model, we considered the use of scoring systems including the *Arbeitsgemeinschaft Gynäkologische Onkologie score* (AGO), peritoneal cancer index (PCI), and peritoneal surface disease severity score (PSDSS), which are all widely used in other cancers, to evaluate the impact of tumor burden without losing reproducibility (14, 15). By integrating prognostic factors including pathological features, surgical factors, and the scoring systems mentioned above, we created a novel prognostic model for OS and PFS in postsurgical EOC patients. To the best of our knowledge, this is the first externally validated nomogram to specifically estimate OS and PFS in EOC patients after R0 reduction surgery.

## Materials and Methods

### Patients

**Figure 1:**
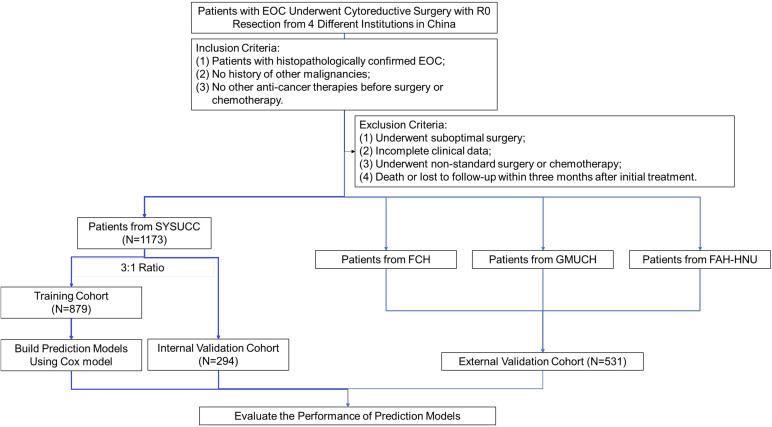
Flowchart of the patient selection process.

From January 2007 to December 2018, medical records of all patients who underwent debulking surgery in our center were comprehensively reviewed and evaluated by gynecologists. The inclusion criteria were as follows: 1. histopathologically confirmed EOC; 2. no history of other malignancies; 3. no other anti-cancer therapies before surgery or neoadjuvant chemotherapy. Patients were excluded if they met any of the following exclusion criteria: 1. suboptimal surgery; 2. incomplete clinical data; 3. non-standard surgery or non-standard first-line chemotherapy; 4. early-stage EOC patients who underwent fertility-saving procedures; 5. previous hysterectomy or unilateral/bilateral salpingo-oophorectomy before diagnosis (e.g., previous treatment of hysteromyoma or ectopic pregnancy); 6. operational records were not detailed enough to calculate PCI score, PSDSS, adjusted AGO score, or surgical complexity score (SCS); 7. death or loss to follow-up within 3 months after initial treatment. After evaluation, a total of 1173 patients were included in this study and were randomly allocated into a derivation cohort of 879 patients and an internal validation cohort of 294 patients, in a ratio of 3:1. Five hundred and seventy-seven patients from another three cancer centers served as the external validation cohort. This retrospective study was approved by the Medical Ethics Committee of all institutions involved and was conducted under relevant guidelines. The workflow is summarized in **Figure 1**.

### Data Collection and Definition

Factors that might impact OS and PFS were extracted and analyzed. Demographic characteristics including age, Eastern Cooperative Oncology Group performance, body mass index, hemoglobin and platelet counts, and neoadjuvant chemotherapy (NACT) status were acquired. Inflammatory data, including leukocytes counts or neutrophil and monocyte percentages, were collected. Tumor characteristics (pathological diagnosis and differentiation, maximum tumor diameter, presurgical CA125 and HE4s, ascites cytology, etc.) were obtained, as well as surgical information including the duration of surgery and amount of blood loss. Biochemical indexes including but not limited to ALT, AST, GGT, TP, ALB, CREA, PT, INR, and APTT, were measured.

We used PCI score and PSDSS to represent the level of tumor burden observed during surgery, as both of these scoring systems are objective, implementable, and frequently used in other malignancies (15–18). PCI score was calculated by assessing the extent of intra-peritoneal disease at diagnosis through quantitatively combining the cancer implant size with the tumor distribution throughout 13 abdominopelvic regions, producing a maximum score of 393. PSDSS was calculated by integrating clinical symptoms, the extent of carcinomatosis, and primary tumor pathology as reported by Antonio et al. (15). We also consulted the settings of the AGO score by utilizing three vital factors to assess the gross disease of patients (complete cytoreduction at initial surgery, no ascites, and good performance status at recurrence). As the AGO score was created for evaluation of secondary cytoreduction, this study used an adjusted AGO score, defined based on complete cytoreduction, no ascites, and good performance status at initial surgery. SCS was used to describe the complexity of the cytoreductive surgery (19, 20). The score sheet used for calculating PCI score, PSDSS, adjusted AGO score, and SCS is summarized in **Supplementary Figure 1**.

### Treatment and Follow-Up

For patients with comorbidity, poor performance status, and predicted suboptimal debulking surgery, neoadjuvant chemotherapy was performed before initial surgery. The possibility of suboptimal resection in the primary debulking setting was assessed by at least two experienced gynecological oncologists and one radiologist. All patients who underwent NACT were assessed with computed tomography (CT) and had Suidan score ≥3. Before NACT was initiated, fine needle biopsy (only for those whose clinical conditions couldn’t tolerate a diagnostic laparoscopy) or diagnostic laparoscopy was completed for histologic confirmation of diagnosis. For those who received interval debulking surgery, surgeries were performed after < 4 cycles of NACT. After surgery, adjuvant chemotherapy was performed when clinically indicated, according to the standard of care based on NCCN guidelines. All patients involved in this research received taxane/platinum-based systemic chemotherapy. Those who did not complete initial treatment or switched to a non-standard regimen were excluded from this study.

All surgeries were performed by experienced gynecological oncologists. For patients in the initial stages (FIGO stages I–II), comprehensive surgical staging was performed, with standard surgical treatment consisting of hysterectomy, bilateral salpingo-oophorectomy, omentectomy, and retroperitoneal (pelvic and para-aortic) lymphadenectomy. For patients with advanced disease (FIGO stages III–IV), cytoreductive surgeries were performed. Surgical approaches included but were not limited to the operations mentioned above, together with other tumorectomies of metastatic lesions if applicable, in an attempt to reach a state of no gross residual disease (complete surgical resection). Peritoneal washing was routinely conducted for all patients.

Patients were followed up every 3 months for the first 2 years after complete initial surgery and adjuvant chemotherapy. After 2 years, patients were followed up annually. Each follow-up consisted of a blood test to determine CA125 and HE4 levels, biochemical tests, and imaging such as abdominal ultrasound or contrast-enhanced CT/magnetic resonance imaging. Recurrence was defined as a persistent elevation in CA125 level or metastases detected by CT/ultrasound. Recurrent EOC was treated by further surgical resection, radiofrequency ablation, or chemotherapy according to the recurrence pattern. The end of follow-up was May 2021. The median follow-up period was 41 months (range: 6.0–160.7 months) for the cohort of patients from our center and 40 months (range: 6.0–149.5 months) for the external validation cohort.

### Statistical Analysis

The endpoints of this study were PFS and OS. PFS was calculated as the interval between the last day of initial treatment and the date when a recurrence was confirmed. OS was defined as the period between the last day of initial treatment and the date of the patient’s death or the date of the last follow-up. Kruskal–Wallis analysis of variance (ANOVA) tests and ANOVA t-tests were performed to compare continuous data. For discrete data, χ^2^-tests were carried out. Survival was calculated by the classical Kaplan–Meier method and compared between groups by log-rank tests. Variables screened as significant by univariate analysis were used in multivariate Cox proportional hazards regression analysis to identify independent prognostic factors.

Based on the multivariate analysis of the training cohort, two nomograms were established. Final model selection was performed by a backward step-down process with the Akaike information criterion. The two nomograms were assessed by Harrell’s concordance index (C-index) and calibration curves comparing model-predicted vs real-world Kaplan–Meier estimates of OS and PFS.

Nomograms were then validated in the internal and external validation cohorts. The nomograms were also compared with the FIGO staging system by analysis of receiver operating characteristic (ROC) curves. SPSS (version 22.0, IBM, Armonk, NY) and R (version 3.6.3) software was used during those procedures. P < 0.05 was defined as statistically significant.

## Results

### Baseline Characteristics of Patients

This multicenter study recruited 1750 patients with a median age of 52.0 (interquartile range: 45–60) years. Of these patients, 630 patients underwent interval debulking surgery (36.0%), whereas others received primary debulking surgery (74.0%). The clinicopathological data of the patients in the training and validation cohorts, including demographic factors, imaging results, surgical factors, and preoperative blood test results, are summarized in **Supplementary Table 1**.

### Independent Prognostic Factors in the Training Cohort

**Figure 2:**
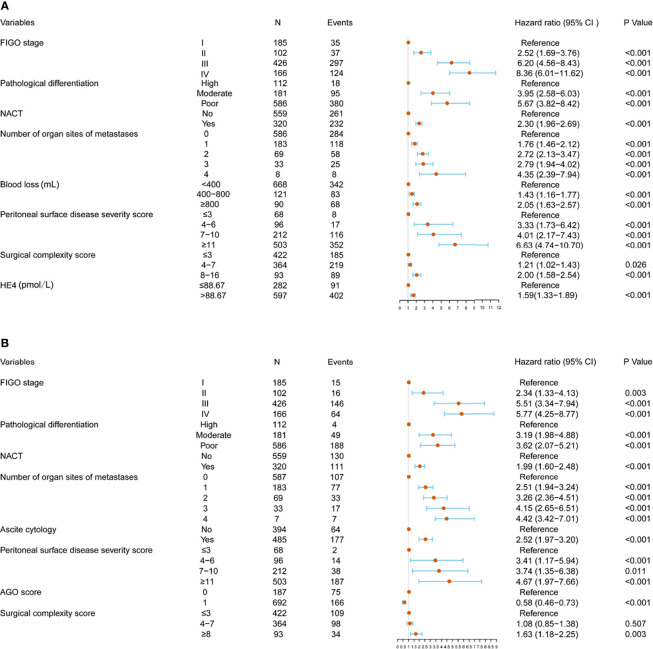
Multivariate analysis and forest plot of the hazard ratios for progression-free survival (PFS) **(A)** and overall survival (OS) **(B)** in the training cohort.

Univariable and multivariable analyses were performed in the training cohort. All significant factors (P < 0.05) in the univariable analysis were entered into the multivariable analysis *via* a Cox regression model. The results showed that FIGO stage, pathological differentiation, NACT, number of organ sites of metastases, blood loss, PSDSS, SCS, and HE4 level were independent risk factors associated with PFS, whereas FIGO stage, pathological differentiation, NACT status, number of organ sites of metastases, ascites cytology, PSDSS, adjusted AGO score, and SCS were independent risk factors associated with OS (**Figure 2**).

### Construction of PFS and OS Predictive Nomograms

**Figure 3:**
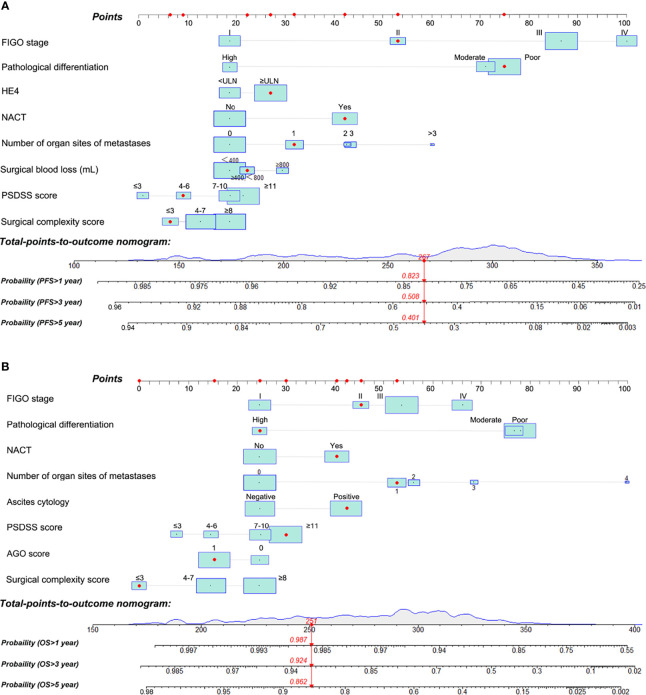
Nomograms for predicting the probability of 1, 3, and 5-year progression-free survival (PFS) **(A)** and 1, 3, and 5-year overall survival (OS) **(B)** of patients with epithelial ovarian cancer. PSDSS, peritoneal surface disease severity score.

To predict OS and PFS of patients with EOC, two nomograms were formulated using R software based on the findings of the multivariable analysis (**Figure 3**). Each value of these variables was assigned as a subtype, therefore a score on the point scale. By adding up the scores for each variable and positioning it on the total point scale, a user can predict the PFS and OS of an individual patient. Red points in the graph represent the values of an observed case. The size of the green boxes represents the proportion of this type of patient in the training cohort, and the density represents the distribution of the variable in the data set.

### Internal and External Validation of Predictive Accuracy

**Figure 4:**
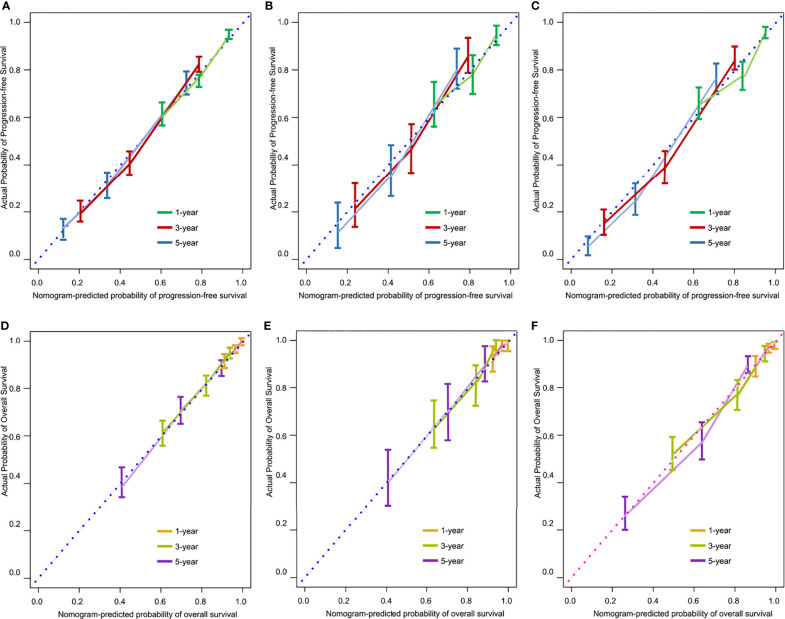
Calibration curves for predicting 1-, 3-, and 5-year progression-free survival (PFS) in the training **(A)**, internal validation **(B)**, and external validation **(C)** cohorts, and for predicting 1-, 3-, and 5-year overall survival (OS) in the training **(D)**, internal validation **(E)**, and external validation **(F)** cohorts.

The Harrell’s C-index values for the PFS and OS nomograms in the training cohort were 0.82 (95% CI: 0.76–0.88) and 0.84 (95% CI: 0.78–0.90), respectively. In the internal validation cohort, the C-index values for the PFS and OS nomograms were 0.80 (95% CI: 0.74–0.86) and 0.81 (95% CI: 0.75–0.87), respectively. In the external validation cohort, the C-index values for the PFS and OS nomograms were 0.84 (95% CI: 0.79–0.89) and 0.87 (95% CI: 0.82–0.92), respectively. The calibration plots showed remarkable agreement between the nomograms’ predictions and actual observations for 1-, 3-, and 5-year PFS and OS in the training cohort (**Figures 4A–D**), internal validation cohort (**Figures 4B–E**), and external validation cohort (**Figures 4C–F**).

### Comparison of Predictive Powers of Nomograms With Conventional FIGO Staging System

**Figure 5:**
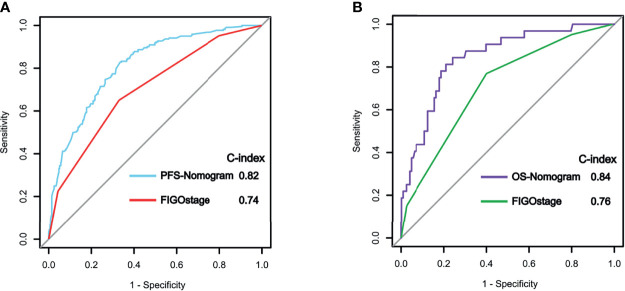
Receiver operating characteristic (ROC) curve analysis of progression-free survival (PFS) **(A)** and overall survival (OS) **(B)** in the training cohort using the nomograms and the FIGO staging system.

We compared the predictive abilities of the PFS and OS nomograms with those of the FIGO staging system using ROC curve analysis. Our nomograms showed better discriminatory power in the training cohort than did the conventional FIGO staging system (**Figure 5**). For the PFS nomogram, the C-index value was 0.82 (95% CI: 0.76–0.88), substantially higher than that of the FIGO staging system (0.74, 95% CI: 0.71–0.81; P < 0.001). For the OS nomogram, the C-index value was 0.84 (95% CI: 0.78–0.90), significantly higher than that of the FIGO staging system (0.76, 95% CI: 0.70–0.78; P < 0.001).

### Nomogram Scores Could Clearly Classify Patients Into Subgroups With Different Risks of Recurrence or Postoperative Mortality

**Figure 6:**
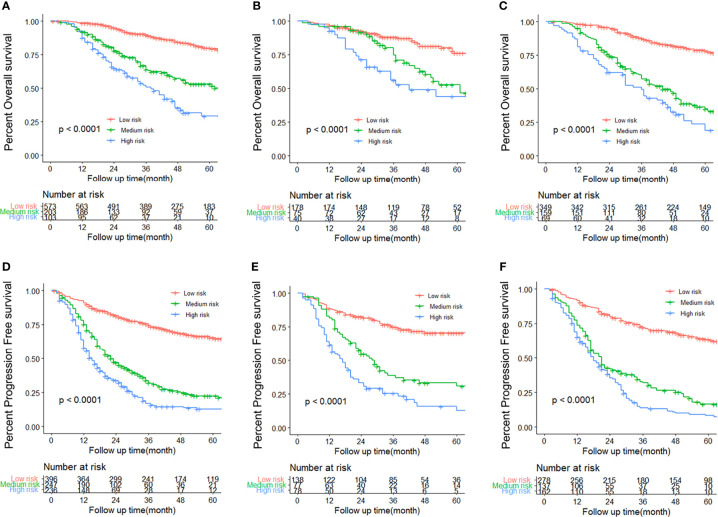
Kaplan–Meier survival curves for each subtype of patients, estimating progression-free survival (PFS) in the training **(A)**, internal validation **(B)**, and external validation **(C)** cohorts, and overall survival (OS) in the training **(D)**, internal validation **(E)**, and external validation **(F)** cohorts.

We determined appropriate cutoff values by grouping the patients in the training cohort evenly into three subgroups after sorting by total score. The OS nomogram could also stratify patients into low risk (score ≤ 172), medium risk (score 172 to 220), and high risk (score > 220) groups. The 5-year PFS rates were 87.4%, 53.2%, and 27.5%, respectively, in the training cohort (P < 0.001) (**Figure 6A**). Similar results were obtained in the internal validation cohort (**Figure 6B**) and in the external validation cohort (**Figure 6C**). Similarly, the PFS nomogram was able to accurately stratify patients as low risk (score ≤ 172), medium risk (score 173-208), or high risk (score > 208) of recurrence, where each group represented a distinct prognosis (**Figure 6D**). The 5-year PFS rates were 71.1%, 25.1%, and 13.6%, respectively, in the training cohort (P < 0.001). Similar results were obtained in the internal validation cohort (**Figure 6E**) and the external validation cohort (**Figure 6F**). While stratifying patients into different risk groups, we took the nomogram’s score of patients from training group into the Cox-model, to explore the relationship between the nomogram score and the probability of >5-year PFS/OS. Using nomogram score as the horizontal axis, and the probability of >5-year PFS/OS as the vertical axis, we found that those two variables are almost linear (**Supplementary Figure 3**).

### Development of Webserver for Easy Access to the Novel Nomograms

The online versions of our nomograms are available for public use; clinicians and researchers can visit https://eocnomogram.shinyapps.io/eocpfs/ and https://eocnomogram.shinyapps.io/eocos/ to predict the long-term survival of postoperative patients with EOC. Predicted survival probability across time can be easily determined by inputting clinical features and reading the output figures and tables generated by the webserver (**Supplementary Figure 2**).

## Discussion

To the best of our knowledge, no prognostic model has previously been constructed specifically for EOC patients undergoing optimal resections. We chose optimal resections as our study interest for the following reasons. Foremost, an unsuccessful cytoreduction offers no appreciable survival benefit and may place patients at risk of morbidity. Efforts are ongoing to better anticipate which patients will experience R1 surgery (21–23). Although there is currently no universally recognized method, there is a growing move towards NACT for patients whose disease is determined to be unresectable (24, 25). In light of this, the prediction of optimal resections with or without NACT is of greater clinical expectations than those underwent R1 surgery. Furthermore, to maximize tumor removal, extensive resections including digestive tract resections or abdominal organ resections may be performed. Therefore, it is natural to hypothesize that “optimal resections” include great disparities that could affect OS and PFS (9, 26). Last, postoperative tumor burden profoundly influences patient prognosis, affecting recurrence and survival (27, 28). Despite optimal resection (residual disease ≤1 cm) being the threshold for satisfactory cytoreduction, various studies have shown that removal of all evidence of macroscopic disease is associated with prolonged survival (5). Hence, the state between complete cytoreductive surgeries and optimal resection is in need of refinement (29).

In the present study, we constructed a PFS-nomogram and OS-nomogram to predict postoperative recurrence and OS in patients who had undergone optimal resection based on conventional clinicopathological and surgical variables. The nomograms showed excellent performance in predicting post-operative PFS and OS of an individual who had undergone optimal resection for EOC. Compared with the conventional FIGO staging system, our nomograms achieved better prediction of outcomes.

Many factors potentially affect the prognosis of EOC patients, including demographic characteristics, pathological diagnosis, imaging results, surgical factors, the volume of residual disease, and the results of relevant blood examinations (30–33). In order to establish a reasonable and accurate model for prediction, all these aspects should be taken into consideration. Unfortunately, there is no prediction model for EOC that attempts to integrate all these types of factors. There is also a conundrum regarding how to describe total tumor burden in a quantified manner. To solve this, we used PCI score and PSDSS to represent the level of tumor burden, as these scoring systems are objective, implementable, and extensively used in malignancies including gastric cancer and colorectal cancer (15–18). In this study, we collected variables that fully covered demographic characteristics (age), pathological diagnosis (FIGO stage, histology, histologic differentiation, ascites cytology, and lymph node invasion), imaging results (Suidan score, largest tumor size by image), surgical factors (operative time, blood loss, volume of ascites, SCS), the tumor burden (PCI score, PSDSS), results of relevant blood tests (levels of HE4, CA125, CA199, hemoglobin, platelets, WBC, RBC, ALT, AST, TP, ALB, SCr, PT, APTT, TBIL, etc.), and other indexes (adjusted AGO score and NACT status). Using univariate and multivariate analysis, we identified risk factors that were associated with PFS and OS. Our finding that PSDSS and SCS were as independent predictive factors for OS and PFS echoes findings from prior studies with significantly larger sample sizes (3, 19, 34). The results validated our speculation that patients who underwent optimal resection with lower presurgical tumor burden, and thus with lower PCI score and PSDSS, might have better prognosis. We established nomograms based on factors including FIGO stage, pathological differentiation, NACT, number of organ sites of metastases, blood loss, PSDSS, SCS, HE4, ascites cytology, and adjusted AGO score, which are strongly associated with PFS and OS.

The two nomograms were validated in a separate internal validation cohort of patients from our center and an external validation cohort of patients from another three cancer centers in different geographic areas in mainland China. The C-index values in the two validation cohorts were 0.80 and 0.85 for predicting PFS and 0.85 and 0.89 for predicting OS. The calibration plots showed good agreement of actual and nomogram-predicted probabilities for PFS and OS in both the internal validation cohort and the external validation cohort. Most previously reported nomograms have lacked external validation, especially multicenter validation (**Supplementary Table 2**) (7, 8, 35–40). According to our multicenter external validation data, the proposed nomograms showed good predictive performance in patients from different areas of China. Thus, they are suitable for national application in clinical practice.

The proposed nomograms could divide postoperative patients into three subgroups with different risks of recurrence or mortality (**Figure 6**). Patients with EOC are often diagnosed at an advanced stage, leading to high rates of mortality and recurrence. Thus, there is a need to improve clinical risk stratification to identify which patients require close surveillance protocols and to design postoperative therapeutic clinical trials. For those with a high risk of recurrence, i.e., patients whose OS-nomogram scores exceeded 220 or PFS-nomogram scores were higher than 208, higher-frequency personalized surveillance programs, hyperthermic intraperitoneal chemotherapy, or targeted therapies might be considered (41).

There were some limitations of this study. The discovery of the BRCA gene and PARP inhibitors heralded a new era for the precision treatment of EOC (42, 43). Unfortunately, as China remains a developing country, most of our patients had not undergone BRCA1/2 gene mutation testing or homologous recombination deficiency testing (HRD). Therefore, there was a lack of information on whether patients in our study harbored genetic mutations. The study also had limitations due to its retrospective nature. For example, the reliability of the PSDSS depends on the validity of surgical records. Future prospective studies are needed to verify our findings.

## Conclusion

An externally validated nomogram predicting OS and PFS in patients after R0 reduction surgery was established. The nomogram score could clearly classify the patients into subgroups with different risks of recurrence or postoperative mortality. The online versions of our nomograms are available at https://eocnomogram.shinyapps.io/eocpfs/ and https://eocnomogram.shinyapps.io/eocos/ . These nomograms may be useful for estimating individual recurrence risk and guiding personalized surveillance programs for patients after surgery, and may potentially open up more options for clinical decision-making or stratification for clinical trials.

## Data Availability Statement

The raw data supporting the conclusions of this article will be made available by the authors, without undue reservation.

## Ethics Statement

The studies involving human participants were reviewed and approved by SYSUCC. The patients/participants provided their written informed consent to participate in this study.

## Author Contributions

QL designed the model and wrote the manuscript with input from all authors. YD analyzed the data and performed the calculations. WW and FY collected and reviewed patient data from the derivation cohort and internal validation cohort. AL, YD, and DY collected and reviewed patient data from the external validation cohort. JL and XZ conceived the study and were in charge of overall direction and planning. QL, YD, WW, FY, AL, DY, XZ, and JL contributed to the interpretation of the results. All authors provided critical feedback and helped to shape the research, analysis, and manuscript.

## Conflict of Interest

The authors declare that the research was conducted in the absence of any commercial or financial relationships that could be construed as a potential conflict of interest.

## Publisher’s Note

All claims expressed in this article are solely those of the authors and do not necessarily represent those of their affiliated organizations, or those of the publisher, the editors and the reviewers. Any product that may be evaluated in this article, or claim that may be made by its manufacturer, is not guaranteed or endorsed by the publisher.

## Supplementary Material

Supplementary Figure 1 Score sheets for calculating PCI (peritoneal cancer index) score **(A)**, peritoneal surface disease severity score (PSDSS) **(B)**, surgical complexity score (SCS) **(C)** and adjusted Arbeitsgemeinschaft Gynäkologische Onkologie (AGO) score **(D)**.

Supplementary Figure 2 Screenshot of the online prediction tool based on the progression-free survival (PFS) **(A)** and overall survival (OS) **(B)** nomogram models.
