# Supplementary figures and images for: Development and External Validation of a Novel Model for Predicting Postsurgical Recurrence and Overall Survival After Cytoreductive R0 Resection of Epithelial Ovarian Cancer
Source: Front Oncol. 2022 Mar 23;12:859409. doi: 10.3389/fonc.2022.859409 (PMC8984120; doi:10.3389/fonc.2022.859409)

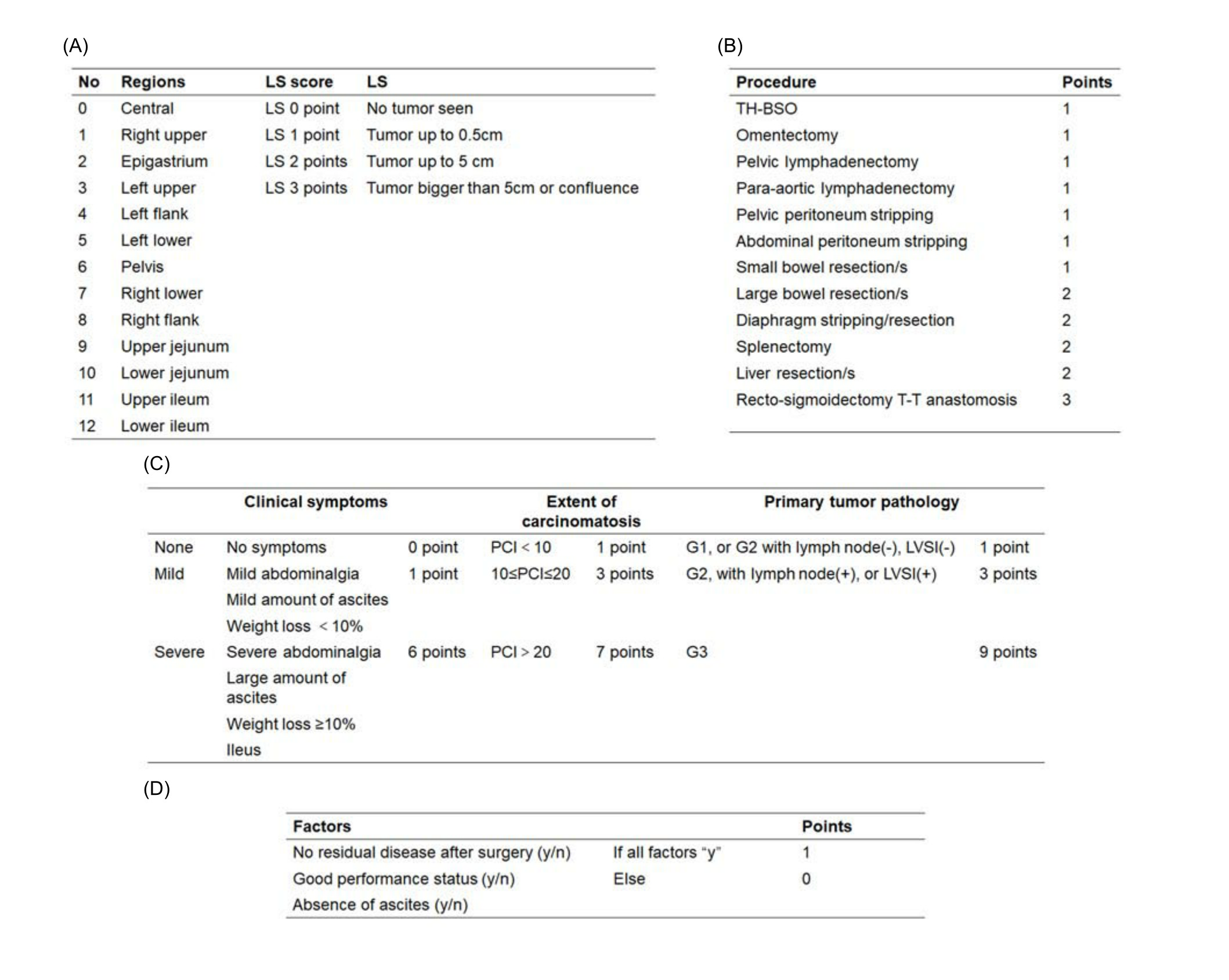

Supplement: Supplementary Figure 1 — Score sheets for calculating PCI (peritoneal cancer index) score (A), peritoneal surface disease severity score (PSDSS) (B), surgical complexity score (SCS) (C) and adjusted Arbeitsgemeinschaft Gynäkologische Onkologie (AGO) score (D). [file Image_1.tif]

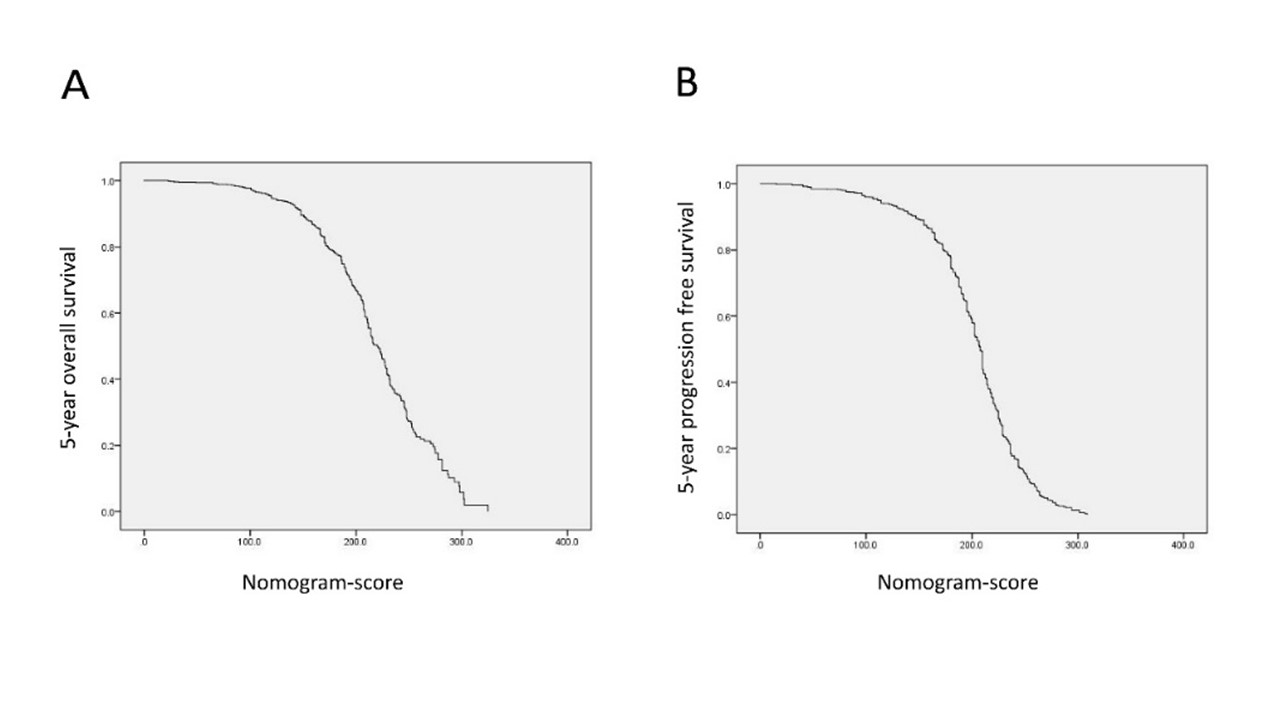

Supplement: Supplementary file 3 [file Image_3.tiff]
