# Supplementary material for: Development and External Validation of a Novel Model for Predicting Postsurgical Recurrence and Overall Survival After Cytoreductive R0 Resection of Epithelial Ovarian Cancer
Source: Front Oncol. 2022 Mar 23;12:859409. doi: 10.3389/fonc.2022.859409 (PMC8984120; doi:10.3389/fonc.2022.859409)
Supplement: Supplementary file 4 [file Table_1.docx]

Supplementary table 1. Baseline characteristics of the three cohorts of patients.

| **Category** | **Variables** | | **Median (interquartile range) or number (proportion, %)** | | | |
| --- | --- | --- | --- | --- | --- | --- |
|  |  |  | **Training cohort (N=879)** | **Internal validation (N=294)** | **External validation (N=577)** | ***P* value** |
|  | Age (years) | | 52(45-60) | 52(45-59) | 53(46-60) | 0.25 |
|  | NACT | | 320(36.4) | 106(36.1) | 204(35.4) | 0.89 |
| **Tumor factors** | FIGO stage | I | 185(21.0) | 68(23.1) | 147(25.4) | 0.62 |
|  |  | II | 102(11.6) | 35(11.9) | 61(10.6) |  |
|  |  | III | 426(48.5) | 137(46.6) | 271(47.0) |  |
|  |  | IV | 166(18.9) | 54(18.4) | 98(17.0) |  |
|  | Histology | Serous | 653(74.3) | 209(71.1) | 418(72.4) | 0.29 |
|  |  | Mucinous | 82(9.3) | 31(10.5) | 48(8.31) |  |
|  |  | Clear cell | 70(8.0) | 26(8.8) | 60(10.4) |  |
|  |  | Endometrioid | 56(6.4) | 24(8.2) | 32(5.5) |  |
|  |  | Mixed/others | 18(2.0) | 4(1.4) | 19(3.2) |  |
|  | Histologic differentiation | Low | 586(66.7) | 193(65.6) | 418(72.4) | 0.06 |
|  |  | Medium | 181(20.6) | 56(19.1) | 89(15.4) |  |
|  |  | High | 112(12.7) | 45(15.3) | 70(12.1) |  |
|  | CA125＞35.0IU/mL | | 695(79.1) | 232(78.9) | 477(82.7) | 0.19 |
|  | HE4＞88.67pmol/L | | 597(67.9) | 201(68.4) | 368(63.8) | 0.20 |
| **Imaging factors** | Suidan score | 0-2 | 657(74.7) | 231(78.6) | 409(70.9) | 0.09 |
|  |  | 3-5 | 196(22.3) | 52(17.7) | 147(25.5) |  |
|  |  | ≥6 | 26(3.0) | 11(3.7) | 14(2.4) |  |
|  | Largest tumor size | ＜5cm | 297(33.8) | 97(33.0) | 157(27.2) | 0.20 |
|  |  | ≥5cm,＜10cm | 298(33.9) | 98(33.3) | 178(30.8) |  |
|  |  | ≥10cm | 284(32.3) | 99(33.7) | 242(41.9) |  |
| **Surgical factors** | Operative time | ＜3h | 290(33.0) | 87(29.6) | 193(33.4) | 0.18 |
|  |  | ≥3h,＜6h | 498(56.7) | 176(59.9) | 306(53.0) |  |
|  |  | ≥6h,＜9h | 83(9.4) | 25(8.5) | 66(11.4) |  |
|  |  | ≥9h | 8(0.90) | 6(2.0) | 12(2.0) |  |
|  | Blood loss | ＜400mL | 668(76.0) | 214(72.8) | 414(71.8) | 0.28 |
|  |  | ≥400mL,＜800mL | 121(13.8) | 50(17.0) | 103(17.9) |  |
|  |  | ≥800mL | 90(10.2) | 30(10.2) | 60(10.4) |  |
|  | Ascites | ＜200mL | 587(66.8) | 196(66.8) | 342(59.3) | 0.16 |
|  |  | ≥200mL,＜1000mL | 173(19.6) | 58(19.7) | 140(24.3) |  |
|  |  | ≥1000mL,＜1500mL | 29(3.3) | 11(3.7) | 20(3.5) |  |
|  |  | ≥1500mL,＜2000mL | 11(1.3) | 3(1.0) | 5(0.8) |  |
|  |  | ≥2000mL | 79(9.0) | 26(8.8) | 70(12.1) |  |
|  | Ascites cytology positive | | 483(54.9) | 154(52.4) | 311(53.9) | 0.74 |
|  | Lymph nodes invasion | | 220(25.0) | 73(24.8) | 157(27.2) | 0.60 |
|  | PCI score | ≤8 | 695(79.0) | 227(77.2) | 462(80.0) | 0.34 |
|  |  | ≥9, ≤16 | 148(16.8) | 48(16.4) | 82(14.2) |  |
|  |  | ≥17 | 36(4.2) | 19(6.4) | 30(5.2) |  |
|  | PSDSS score | ≤3 | 68(7.7) | 20(6.8) | 58(10.1) | 0.37 |
|  |  | ≥4, ≤6 | 96(10.9) | 30(10.2) | 48(8.3) |  |
|  |  | ≥7, ≤10 | 212(24.1) | 79(26.8) | 140(24.3) |  |
|  |  | ≥11 | 503(57.3) | 164(55.8) | 331(57.4) |  |
|  | AGO score =1 | | 689(78.4) | 233(79.3) | 430(74.5) | 0.15 |
|  | SCS score | ≤3 | 422(48.0) | 144(49.0) | 242(41.9) | 0.09 |
|  |  | ≥4, ≤7 | 364(41.4) | 113(38.4) | 270(46.8) |  |
|  |  | ≥8, ≤11 | 93(10.5) | 37(12.6) | 65(11.3) |  |
| **Preoperative blood examinations** | Hemoglobin (g/L) | | 119.7  (106.2-129.6) | 119.0  (103.3-129.0) | 119.2  (105.1-129.3) | 0.65 |
|  | Platelet (×10^9^/L) | | 255.0  (195.3-328.5) | 251.0  (196.3-327.8) | 250  (191.2-319.6) | 0.57 |
|  | White blood cell (×10^9^/L) | | 6.0(4.7-7.7) | 6.0(4.8-7.8) | 5.9(4.6-7.6) | 0.37 |
|  | Red blood cell (×10^9^/L) | | 4.1(3.7-4.5) | 4.2(3.7-6.4) | 4.1(3.7-4.6) | 0.45 |
|  | NE percentage (%) | | 64.3(55.9-73.3) | 64.0(55.8-72.9) | 63.7(56.2-72.3) | 0.66 |
|  | MO percentage (%) | | 7.0(5.4-8.3) | 7.0(5.2-8.3) | 6.7(5.4-8.3) | 0.41 |
|  | LY percentage (%) | | 27.1(18.9-33.9) | 27.0(19.1-33.4) | 27.1(19.2-33.6) | 0.96 |
|  | ALT (U/L) | | 14.7(10.7-21.1) | 14.3(10.9-20.4) | 14.5(10.7-20.7) | 0.24 |
|  | AST (U/L) | | 18.8(15.3-23.3) | 18.5(15.3-23.2 | 18.6(15.5-23.4) | 0.63 |
|  | GGT (U/L) | | 19.3(13.8-29.1) | 19.6(14.0-28.9) | 19.8(13.7-28.7) | 0.45 |
|  | TP (g/L) | | 72.5(67.5-76.6) | 72.3(68.5-76.7) | 72.1(67.9-76.5) | 0.11 |
|  | ALB (g/L) | | 41.2(38.0-43.6) | 41.7(38.6-43.8) | 41.2(38.2-43.5) | 0.37 |
|  | GLOB (g/L) | | 30.8(27.9-34.3) | 30.6(27.5-34.4) | 30.6(27.8-34.1) | 0.82 |
|  | SCr (μmol/L) | | 55.4(48.5-62.2) | 54.3(48.4-62.7) | 54.9(48.5-62.2) | 0.65 |
|  | PT (sec.) | | 11.5(11.1-12.1) | 11.4(10.9-12.0) | 11.5(11.1-12.1) | 0.69 |
|  | APTT (sec.) | | 26.0(23.4-28.6) | 26.3(23.8-28.5) | 26.1(23.1-28.8) | 0.56 |
|  | INR | | 1.0(0.95-1.1) | 0.99(0.95-1.0) | 1.0(0.96-1.1) | 0.29 |
|  | D-Dimer (μg/mL) | | 1.3(0.59-2.9) | 1.1(0.60-2.6) | 1.1(0.66-2.7) | 0.36 |
|  | TBIL (μmol/L) | | 8.3(6.4-10.5) | 8.4(6.4-10.4) | 8.4(6.5-10.5) | 0.64 |
|  | DBIL (μmol/L) | | 2.5(1.8-3.3) | 2.5(1.9-3.2) | 2.5(1.9-3.4) | 0.72 |
|  | TC (mmol/L) | | 4.9(4.2-5.6) | 5.1(4.4-5.8) | 5.0(4.3-5.6) | 0.09 |
|  | TG (mmol/L) | | 1.3(0.93-1.7) | 1.3(0.89-1.9) | 1.3(0.93-1.8) | 0.50 |

Supplementary table 1. Baseline characteristics of the three cohorts of patients.

NACT, neoadjuvant chemotherapy; FIGO, international federation of gynecology and obstetrics; PCI, peritoneal cancer index; PSDSS, peritoneal surface disease severity score; AGO, Arbeitsgemeinschaft Gynakologische Onkologie; SCS, surgical complexity score; NE, neutrophilic granulocyte; MO, monocytes; LY, lymphocytes; ALT, alanine aminotransferase; AST, aspartate aminotransferase; GGT, glutamyl transpeptidase; TP, total protein; ALB, serum albumin; GLOB, globulin; SCr, serum creatinine; PT, prothrombin time, APTT, activated partial thromboplastin time; INR, international normalized ratio; TBIL, total bilirubin; DBIL, direct bilirubin; TC, serum total cholesterol; TG, triglyceride.
