# Supplementary material for: Development and External Validation of a Novel Model for Predicting Postsurgical Recurrence and Overall Survival After Cytoreductive R0 Resection of Epithelial Ovarian Cancer
Source: Front Oncol. 2022 Mar 23;12:859409. doi: 10.3389/fonc.2022.859409 (PMC8984120; doi:10.3389/fonc.2022.859409)
Supplement: Supplementary file 5 [file Table_2.docx]

Supplementary Table 2. Typical postsurgical OS/PFS predictive nomograms for EOC patients underwent cytoreductive surgery reported in recent literature.

| Author, year | Region | Number of patients | Patient criteria | Outcomes | Variables recruited in nomogram | | | | C-index | | External validation |
| --- | --- | --- | --- | --- | --- | --- | --- | --- | --- | --- | --- |
|  |  |  |  |  | Tumor Factors | Imaging factors | Surgical factors | Blood examinations |  |  |  |
| Barlin, 2012 | USA | 478 | Patients underwent primary surgery | 5-year  disease specific mortality | Stage, histology | None | ASA, debulking status | ALB | 0.714 | No, single center | |
| Wei, 2019 | China | 142 | advanced HGSOC patients | 18 months to 3 years recurrence probability | Ago, FIGO stage, menopause status | Radiomic signature | Residual disease | CA125 | 0.673 | No | |
| Rutten, 2014 | The Netherlands | 840 | primary as well as interval debulking surgery | 1, 3, 5 -year survival | Age, NACT, histology, FIGO stage, BRCA | None | Residual disease, ascites | None | 0.710 | Yes, 3 centers | |
| Chi,2007 | USA | 424 | stage IIIC EOC | 5-year disease specific survival | Age, Grade, Histology | None | Residual disease | PLT count, ascites | 0.670 | No, single institution | |
| Wang, 2021 | SEER database | 9001 | EOC patients | 3-,5-year survival | Race, age, AJCC stage, Lymph nodes status, primary site surgery, radiotherapy | None | None | None | 0.733 | No | |
| Xu, 2017 | SEER database | 10692 | EOC patients | 3-,5-year survival | Marital status, age, race, grade, histology, RLNs, LODDs | None | None | None | 0.699 | No | |
| Yang, 2021 | China | 114 | EOC | 1-,2-year recurrence | FIGO stage, Pathological grade, Lymph nodes, ascites | None | None | M-CTC percentage, CTC counts, CA125 | 0.913 | Yes, 2 centers | |
| The present study | China | 1173 | EOC | 1-, 3, 5-year recurrence | FIGO stage, pathological differentiation, NACT status, ascites cytology | None | PSDSS score, SCS score, blood loss, | HE4 | 0.82 | Yes, 4 centers | |
